# Use of a disposable vascular pressure device to guide balloon inflation of resuscitative endovascular balloon occlusion of the aorta: a bench study

**DOI:** 10.1038/s41598-021-03502-6

**Published:** 2021-12-15

**Authors:** Anja Levis, Nives Egli, Hansjoerg Jenni, Wolf E. Hautz, James I. Daley, Matthias Haenggi

**Affiliations:** 1 Department of Anesthesiology and Pain Medicine, Inselspital, Bern University Hospital, University of Bern, Bern, Switzerland; 2 Department of Intensive Care Medicine, Inselspital, Bern University Hospital, University of Bern, Bern, Switzerland; 3 Department of Cardiovascular Surgery, Inselspital, Bern University Hospital, University of Bern, Bern, Switzerland; 4 Department of Emergency Medicine, Inselspital, Bern University Hospital, University of Bern, Bern, Switzerland; 5 Department of Emergency Medicine, Yale University School of Medicine, New Haven, CT USA

**Keywords:** Acute coronary syndromes, Arrhythmias, Trauma, Preclinical research

## Abstract

Resuscitative endovascular balloon occlusion of the aorta (REBOA) for rapid hemorrhage control is increasingly being used in trauma management. Its beneficial hemodynamic effects on unstable patients beyond temporal hemostasis has led to growing interest in its use in other patient populations, such as during cardiac arrest from nontraumatic causes. The ability to insert the catheters without fluoroscopic guidance makes the technique available in the prehospital setting. However, in addition to correct positioning, challenges include reliably achieving aortic occlusion while minimizing the risk of balloon rupture. Without fluoroscopic control, inflation of the balloon relies on estimated aortic diameters and on the disappearing pulse in the contralateral femoral artery. In the case of cardiac arrest or absent palpable pulses, balloon inflation is associated with excess risk of overinflation and adverse events (vessel damage, balloon rupture). In this bench study, we examined how the pressure in the balloon is related to the surrounding blood pressure and the balloon's contact with the vessel wall in two sets of experiments, including a pulsatile circulation model. With this data, we developed a rule of thumb to guide balloon inflation of the ER-REBOA catheter with a simple disposable pressure-reading device (COMPASS). We recommend slowly filling the balloon with saline until the measured balloon pressure is 160 mmHg, or 16 mL of saline have been used. If after 16 mL the balloon pressure is still below 160 mmHg, saline should be added in 1-mL increments, which increases the pressure target about 10 mmHg at each step, until the maximum balloon pressure is reached at 240 mmHg (= 24 mL inflation volume). A balloon pressure greater than 250 mmHg indicates overinflation. With this rule and a disposable pressure-reading device (COMPASS), ER-REBOA balloons can be safely filled in austere environments where fluoroscopy is unavailable. Pressure monitoring of the balloon allows for recognition of unintended deflation or rupture of the balloon.

## Introduction

Exsanguination is a major cause of traumatic cardiac arrest in both civilian^1^ and military^2^ environments. Emergency resuscitative thoracotomy is the recommended approach in certain trauma victims presenting in or at risk of impending cardiac arrest, but this approach is limited to a specific subset of trauma patients^3–5^ presenting in facilities capable of performing this invasive procedure. Although emergency resuscitative thoracotomy can be performed in the prehospital environment^6^, real-world data point toward restricting use of this intervention, even if indicated^7^.

Resuscitative endovascular balloon occlusion of the aorta (REBOA) is a catheter-based technique using a balloon within the descending aorta to occlude the vessel, analogous to an internal cross clamp. Similar to the open approach through thoracotomy, REBOA is a lifesaving intervention designed to temporarily stop the bleeding and stabilize the patient until definitive treatment. REBOA has been successfully used when resuscitative thoracotomy could not be performed in exsanguinating patients in the absence of thoracic trauma^3,8^.

In addition to the obvious benefit of limiting blood loss, occlusion of the descending aorta can have a potentially beneficial hemodynamic effect by centralizing the circulating volume, thereby increasing central blood pressure. Indication for REBOA is expanding to nontraumatic cardiac arrest^9–11^. REBOA can be implemented in the prehospital environment^12–15^, and prehospital REBOA in trauma could add a substantial mortality benefit in selected patients^16^.

Whereas the first-generation catheters for REBOA utilized large 12Fr introducer sheaths that frequently required vessel cut-down for insertion, smaller, second-generation catheters can be inserted through a 7Fr introducer sheath that is much more amenable to placement with the ultrasound-guided Seldinger technique^17^. Common femoral artery (CFA) access using portable ultrasound with Doppler can be performed outside the hospital and in austere environments^12,14^. The volume needed for balloon filling and occlusion of the aorta mainly depends on the aortic diameter of the individual patient in the respective zone (zones 1–3, from intrathoracic to above iliac bifurcation). In the hospital setting, filling of the REBOA balloon can be visualized by fluoroscopy during insertion by inflating the balloon with a mixture of contrast and saline^18,19^. In settings where fluoroscopy or serial x-ray is not available, balloon inflation to aortic occlusion can be guided by palpation or Doppler ultrasound of the contralateral CFA, with observation of the pulsatile arterial curve derived from the introducer sheath, or with inflation with a fixed volume^14^. In patients in cardiac arrest, guidance by palpation of pulse or Doppler ultrasound of the contralateral CFA is not feasible, and monitoring the pressure in the CFA at the side port of the sheath during inflation is not reliable, mainly because pulse pressure generated by chest compressions is transmitted to the CFA^11^. Overinflation of the balloon has led to complications^20^, and during our study in cardiac arrest patients, we encountered one balloon rupture^11^, which has occurred in similar clinical settings^21^. Consequently, a simple and reliable method to guide balloon inflation is needed.

**Figure 1:**
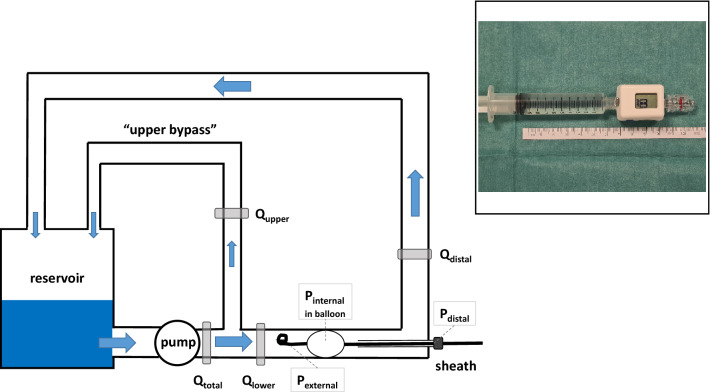
Schematic drawing of the model. Q denotes flow measured on the flow probes (“total” flow in the system, “upper” flow in the bypass resembling the upper body, “lower” in the part resembling the lower body, and “distal” the flow distal to the balloon), and P denotes pressure (“external” the blood pressure at the tip of the REBOA catheter, “internal” the pressure in the balloon of the REBOA catheter, and “distal” the blood pressure at the side port of the sheath). The diameter of the “descending aorta” is 21 mm. The inset shows a photograph of the COMPASS device with a 10 ml syringe attached. The device has two Luer connectors (male/female) for in-line connection in a pressure line and measures about 1.5 × 2 × 3 cm.

The Centurion COMPASS Universal Hg device is a lightweight, disposable, ready-to-use in-line pressure-monitoring system measuring 1.5 × 2 × 3 cm, with two standard Luer connectors, intended to measure the mean arterial blood pressure (Fig. 1, inset). We hypothesized that the inflated REBOA balloon can be used as a transmitter of the aortic blood pressure and therefore measuring the balloon's internal pressure can guide balloon inflation without risk of overinflation. With this bench study, we aimed to evaluate the use of internal balloon pressure monitoring with the COMPASS device to guide the correct inflation of REBOA balloons in settings where fluoroscopy is not available, for example, in the prehospital setting.

## Methods

We performed two sets of bench-test experiments; for clarity, we describe them as static (set 1) and flow (set 2) experiments.

### Materials

We used the ER-REBOA catheter (Prytime Medical, Bourne, TX, USA) in our tests. Pressures were measured with a Biopac MP160 Data Acquisition System and the Biopac MP3X/45 pressure transducer, with Acqknowledge 5.0 software (Biopac Systems, Goleta, CA, USA; https://www.biopac.com/product-category/research/software/). The Biopac pressure transducer resembles a generic pressure transducer used for arterial or central venous pressure monitoring in humans. The data were stored on a standard laptop. In addition, we simultaneously used the Centurion COMPASS (Centurion Medical Products, Williamston, MI, USA), which was connected in-line between the balloon connector and the pressure transducer of the Biopac data acquisition system, to read the internal ER-REBOA balloon pressure. Because the device has no data storage or data output interface, the readings at the end of each measurement period were recorded manually by two experimenters. For ease of calculation, the stored values from the Biopac system were used (which were identical to the manually recorded values).

**Figure 2:**
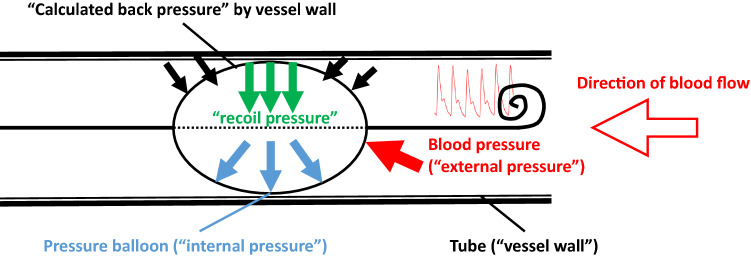
Schematic drawing of forces and resulting pressures. As long as there is no wall contact, the internal pressure in the balloon arises from elastic recoil forces of the balloon and the surrounding, external blood pressure. Once the balloon is wedged in the vessel/tube, the internal balloon pressure results from the elastic recoil forces of the balloon, the external blood pressure, and the calculated back pressure of the surrounding vessel.

We labeled the pressure measured in the balloon “internal pressure,” the pressure of the medium outside the balloon (blood pressure; in our model, water) “external pressure,” the pressure generated by the recoil forces of the balloon “recoil pressure,” and the pressure generated by the vessel wall (in our model, tubing) counteracting the pressure exerted by the balloon “calculated back pressure” (Fig. 2). The back pressure is not directly amenable but can be calculated with the formula calculated back pressure = internal pressure − external pressure − recoil pressure.

For the static experiments (set 1), we used a Perfusor Space syringe pump (B. Braun, Melsungen, Germany) for slow balloon inflation with the pressure recordings described above. Two silicone tubes with a diameter of 21 and 25 mm representing a normal and a large human aorta at diaphragmatic level were used.

For the flow experiments (set 2), a circuit filled with water was built (see Fig. 1 for a schematic view). The pulsatile “heart” of the circuit was a magnetically mounted radial blood pump that is usually used as an extracorporeal assist device, or in combination with an oxygenator, as an extracorporeal membrane oxygenation (ECMO) pump (MEDOS DP3, Xenios, Heilbronn, Germany). The “venous” pool was a standard cardiotomy reservoir (Inspire; LivaNova, London, UK) that is used in cardiovascular surgery as a reservoir for extracorporeal circulation during surgery. The circuit was made of standard extracorporeal circulation (ECC) silicone tubing, with a bypass attached between the pump and the “aorta” to simulate upper body circulation. The “descending aorta” was made of a silicone tube with a diameter of 21 mm. The ER-REBOA catheter was inserted according the instructions for use with a 7Fr sheath (Radifocus Introducer II, Terumo Europe, Spreitenbach, Switzerland). Total flow, distribution between the parts of the circuit representing the upper and lower body (containing the silicone tube representing the descending aorta), and the pulsatile pressure in the system resulted from adaptation of the rotation velocity of the DP3 pump and adjustable clamps on the silicone tubes. Flow was measured using ultrasound flow probes (em-tec, Finning, Germany) usually used in ECMO and in ECC during surgery, which can be clamped around the tubes in the circuit (Fig. 2).

### Protocol

Set 1. Static experiments: To test balloon compliance, the balloon was filled with saline at a rate of 1 mL/min while the catheter was suspended, thereby ensuring the balloon could expand freely while staying at the level of the pressure transducer. Pressure (upper, lower, and mean through the Biopac system, and mean pressure with the COMPASS device) was measured simultaneously at the connector at the balloon filling line. Thus, a curve representing the pressure within the balloon for a filling volume of 0–24 mL was obtained. The balloon outer diameter was measured using a caliper every 2 min, representing an additional 2 mL of saline added into the balloon in the range of 8–20 mL. The experiment was then repeated with the balloon surrounded by the silicone tubes representing a human aorta of 21 mm and 25 mm.

To test balloon compliance under various external pressures, the balloon was placed in an infusion bag containing 3 L of normal saline. A blood pressure cuff was used to apply static pressure of 30 and 90 mmHg to the bag.

Set 2. Flow experiment: The experiment was conducted under dynamic conditions in the circuit described above. Pump speed and resistance were adjusted until a total flow of 3.3 L/min was achieved, divided into 2 L/min to the part containing the silicone tube representing the descending aorta and 1.3 L/min to the upper bypass, and a pressure of approximately 80/30 mmHg. The balloon was again filled with a continuous infusion of saline while we simultaneously measured the pressure in the balloon, at the catheter tip, and distal to the balloon, as well as the flow distal to the balloon and through the upper bypass. Once the balloon started to move as a result of the pulsatile pressure in the system, it was manually held in place to prevent dislocation. To ensure reproducibility, the experiment was repeated three times.

## Results

The mean internal pressures obtained with the COMPASS device were identical to those measured by the transducers. Variations of all pressures between the replicated experiments/runs were generally small and under 10%, which we consider clinically not important for this application. To improve readability, we display only mean values in the figures; detailed results including means and standard deviations and relative standard deviations can be found in the online supplement.

**Figure 3:**
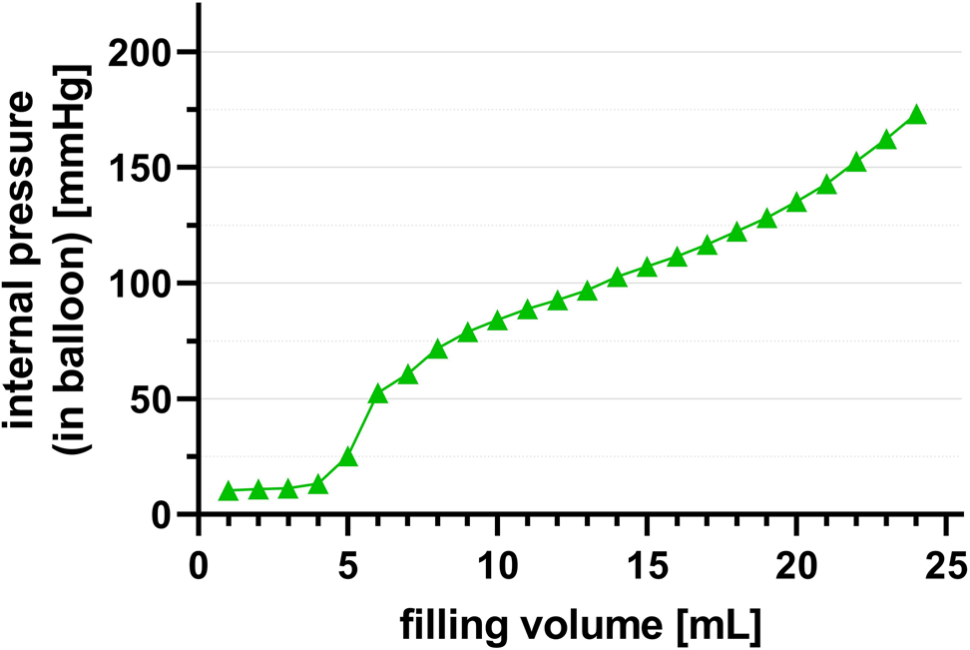
ER-REBOA Compliance/elastance of the balloon when suspended in air. After overcoming initial elastic recoil forces, compliance of balloon remains constant within the recommended volumes.

Set 1. Static experiments: When the apparatus was suspended in air, the internal pressure in the balloon showed an initial steep increase at a filling volume of 5–7 mL, corresponding to the opening pressure to be overcome. Of note, the catheter’s dead space is below 1 mL. Above this filling volume, the compliance curve flattened, becoming almost linear between 8 and 20 mL and becoming steeper again from 20 to 24 mL with a maximal internal pressure of 180 mmHg in the balloon (Fig. 3, and Table 1).

**Table 1:** Derivation of the “rule of thumb”.

Filling volume (mL)	Balloon diameter corresponding to filling volume (mm)	Internal balloon pressure with catheter suspended in air (mmHg)	Addition of an estimated external mean arterial blood pressure (CPR, 40 mmHg)	Addition of the estimated calculated back pressure of the aorta (+ 30 mmHg), = target pressure for total occlusion
5	15	23	63	93
6		47	87	117
7		61	101	131
8	20	70	110	140
9		77	117	147
10		83	123	153
11		88	128	158
12		92	132	162
13	25	97	137	167
14		102	142	172
15		108	148	178
16		112	152	182
17		117	157	187
18		123	163	193
19		129	169	199
20	30	136	176	206
21		144	184	214
22		154	194	224
23		164	204	234
24 (MAX)	32 (MAX)	173	213	243

Columns 1, 2, and 3 show the balloon filling volume, resulting balloon diameter, and the resulting internal pressures when the balloon is suspended in the air and no external pressurized fluid/blood and no back pressure increase the internal balloon pressure. In column 4, we added a hypothetical mean arterial pressure of 40 mmHg, as would be generated by CPR, as the surrounding external pressure. In the 5th column, we added the calculated back pressure of the vessel at full aortic occlusion (+ 30 mmHg). Filling the balloon up to an internal pressure of 160 mmHg during CPR (or another low flow/low pressure state) should occlude most descending aortas. If the pressure of 160 mmHg is not reached at a volume of 16 ml, additional filling in 1-mL steps should increase the pressure by not more than 10 mmHg per step. That is, filling volume (in mL) × 10 = target pressure (in mmHg).

**Figure 4:**
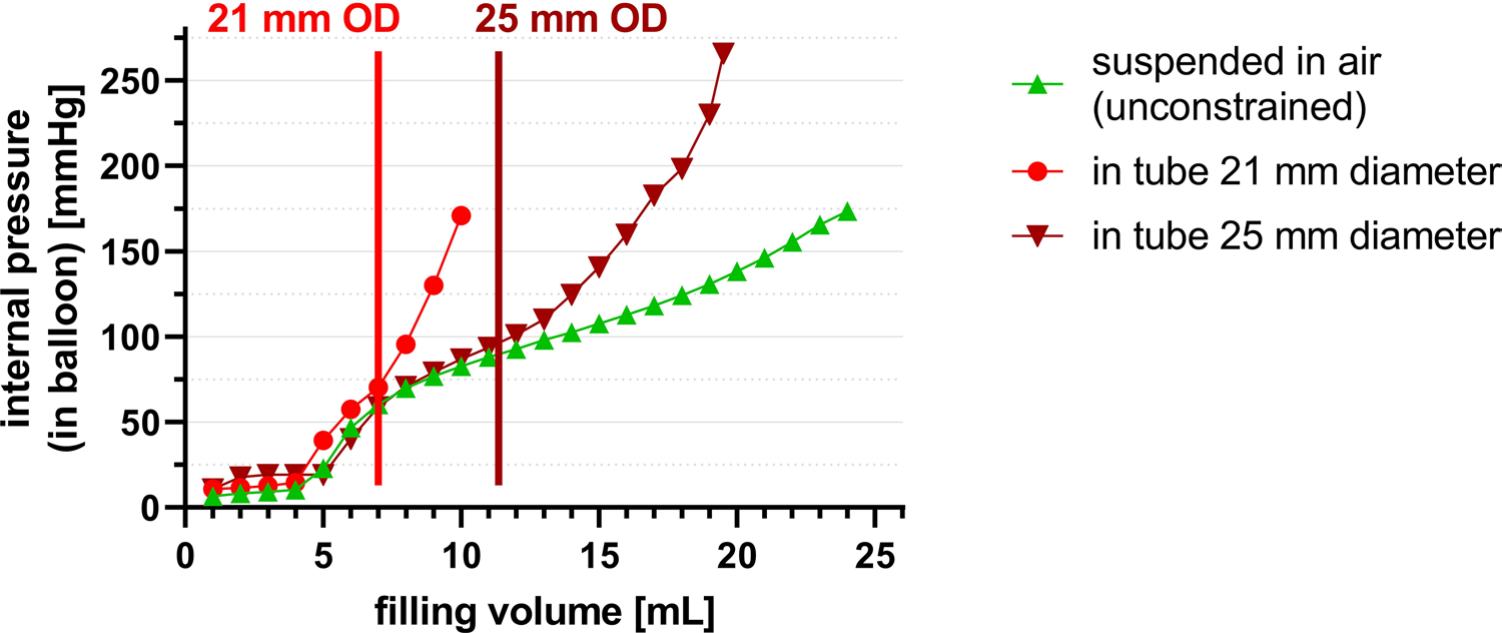
Differences in internal pressure (within the balloon) while inflated in air (unconstrained; green line), or in silicone tubes of 21-mm or 25-mm diameter in static conditions. Once balloon diameter reaches the inner diameter of the tube, internal pressure rises sharply. OD = outer diameter.

When external, static pressure was applied to the 3-L infusion bag containing the balloon, this external pressure was fully transmitted and therefore added to the recoil pressure generated by the balloon filling itself (measured as internal pressure = recoil pressure + external pressure). When the balloon was placed in the tubes of 21 respective 25 mm inner diameter, the pressure–volume curve began to diverge from the balloon compliance curve once the outer diameter of the balloon equaled the inner diameter of the tube (Fig. 4), allowing for calculation of the back pressure (see formula above).

**Figure 5:**
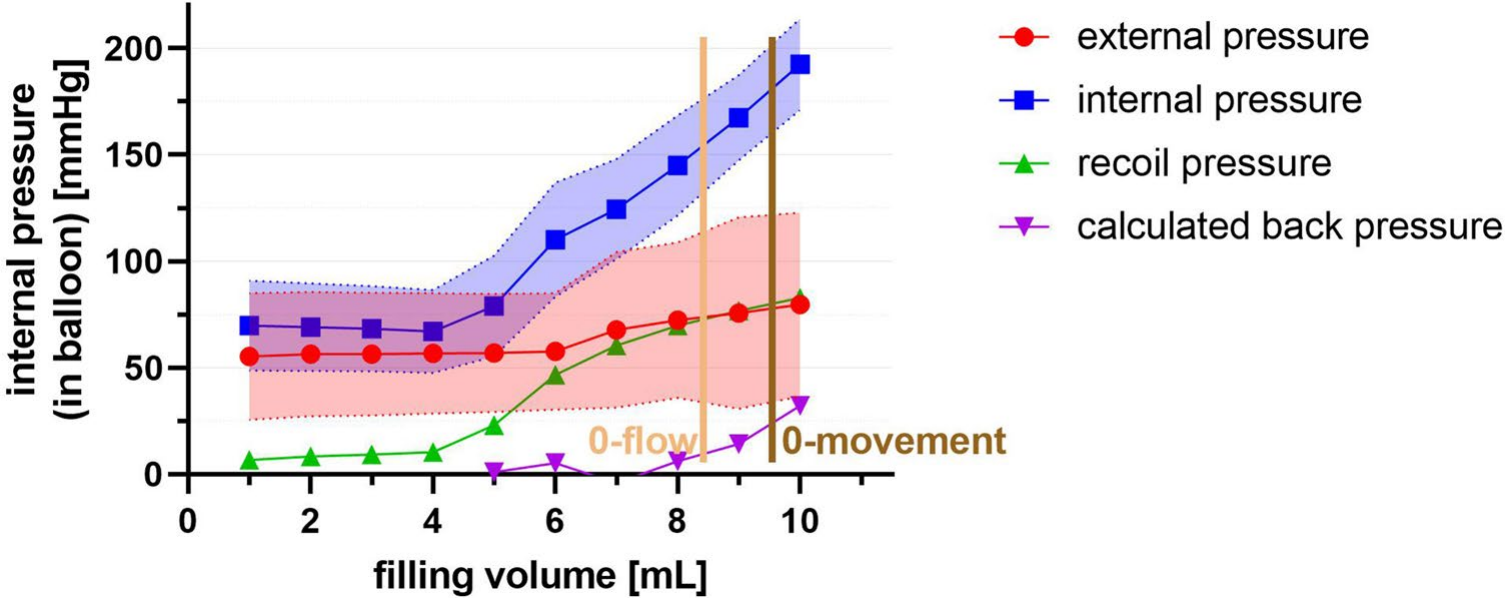
Measured pressures (external, internal, recoil) and calculated back pressure from the vessel wall. Once the balloon is inflated to 21 mm (inner diameter of the tube) at 8 mL filling volume, pressure in the balloon rises without any changes to the surrounding blood pressure, and flow stops (0-flow). Adding 1 mL filling volume increases the pressure enough so that the balloon is wedged in the vessel wall and any pulsatile movement stops (0-movement). Further balloon filling exerts pressure on the vessel wall only, without changes to blood flow.

Set 2. Flow experiment: When a pulsatile pressure was applied, the upper and lower internal pressures obtained in the balloon were similar to those that would be obtained if a static high or static low external pressure had been applied, as long as the balloon did not represent an obstacle to flow. Once the expanding balloon began to affect the flow in the tubing, the increase in internal balloon pressure was a little less pronounced compared to the increase within the static system, most likely because of the pulsatile movements of the balloon (mean difference between filling volumes of 5 mL and 9 mL: -2.4 mmHg, or 2.5%). In a circuit with pulsatile flow, flow distal to the occlusion ceased once the internal pressure in the balloon remained higher than the surrounding pressure (Fig. 5). The calculated back pressure at zero flow is around 10 mmHg.

Because of the cylindrical shape of the balloon, rhythmic rotational movements at the pulse rate occurred until further inflation made the balloon “wedge” completely in the tubing. Calculated back pressure at the moment the movements stopped was between 20 and 25 mmHg (Fig. 5).

## Discussion

In general, the pressure measured within a balloon is determined by its filling volume, its compliance, and its surrounding pressure. If expansion of the balloon is limited by external boundaries, such as a silicone tube or the wall of a vessel, the pressure exerted by this structure on the balloon adds to the pressure within the balloon. Thus, the pressure measured within a balloon (i.e., the internal pressure) can be described as the sum of the pressure arising from the recoil force of the filled balloon (depending on balloon compliance) and the external pressure (blood pressure in the case of a balloon in the aorta, and back pressure arising from the interaction of the balloon with the limiting structure). Once back pressure rises from zero (no contact with vessel wall) to a value high enough to wedge the balloon in the vessel, blood no longer flows across this obstacle. When total aortic occlusion is desired, targeting a threshold back pressure may therefore be helpful. As the pressure between the aortic wall and balloon cannot be measured directly, it has to be estimated from total pressure measured in the balloon. This is possible because the other determinants are known, as described above.

This concepts makes it possible to calculate the internal balloon pressure where, at a given blood pressure, the vessel is occluded, independent of the type and manufacturer of the catheter, and with any transducer or device that can measure pressures. Because in a medical emergency, especially in austere environments, setting up a second traditional pressure transducer and calculations of system compliances are not advisable, we used a stepwise approach to develop a simple “rule-of-thumb” algorithm for the REBOA catheter system used in the city of Bern Emergency Medical System.

The ER-REBOA catheter balloon is designed to have a flat compliance curve for the range of 8–20 mL of filling volume, resulting in an increase of internal pressure within the balloon of less than 10 mmHg for every milliliter of filling volume when the balloon is allowed to freely expand (see Fig. 1). The resulting balloon diameter of 20–30 mm lies within the diameter of the descending aorta.

Once the expanding balloon limits the flow in the vessel, the internal balloon pressure is determined by recoil pressure, external blood pressure, and back pressure from the surrounding structure. A pulsatile pressure swing is still recognizable within the balloon but is negligible in amplitude.

Before the balloon completely occludes the vessel, the flow decreases and the balloon starts to move rhythmically as a result of the force exerted by the pulsatile flow on its surface. Movement of the balloon continues after flow has ceased until the pressure between the balloon and the vessel walls is high enough for the balloon to become wedged in the vessel and to counteract the pressure generated by the pump or heart and transmitted to the balloon via the liquid column.

Given the known compliance curve of the catheter and the findings from our experiment, and further assuming a mean arterial pressure of approximately 40 mmHg during cardiopulmonary resuscitation (CPR; although maximal “systolic” pressures can be much higher^22^), we can advise a simple rule of thumb derived from experimental data and the known diameters of the balloon at any given filling volume (taken form the manufacturer’s instructions for use), which we present in Table 1.

The balloon should be filled until the internal pressure measured in the balloon with the COMPASS device is at least 160 mmHg. If this pressure is reached before a filling volume of 16 mL is applied and the balloon no longer moves rhythmically, occlusion and wedging of the balloon is likely to have occurred. If the balloon is still expanding outward, filling should be continued slowly until movement stops, but without exceeding a maximal pressure of 200–220 mmHg at maximum 16 mL filling volume.

If at 16-mL filling volume the pressure in the balloon has not yet reached 160 mmHg, it is likely that the balloon has not yet made contact with the vessel wall. Filling therefore should be continued, with the pressure in the balloon checked after every added milliliter. For every milliliter added after 16 mL, target pressure in the balloon increases by 10 mmHg. In other words, target pressure in the balloon is 170 mmHg for a filling volume of 17 mL, 180 mmHg for a filling volume of 18 mL, and so on. Filling should be continued until the desired pressure for the respective volume occurs. For safety reasons and to limit vessel wall stress, the pressure in the balloon should not exceed 240–250 mmHg. If the device is still pushed distally despite adequate filling volume and corresponding target pressure being reached, one more milliliter can be added to achieve wedging of the balloon, as long as the safety limit of 240 -250 mmHg is not exceeded. Alternatively, the balloon can be secured in place without this additional milliliter, because distal flow ceases before wedging of the balloon occurs.

With these rules of thumb, full occlusion can be achieved with reduced risk of vessel damage. Additionally, just before reaching the target pressure, the pulsatile movements diminish, which is an additional sign of complete occlusion. During full occlusion, monitoring the balloon pressure can prevent unnoticed balloon rupture, which has been described in trauma resuscitation^21^ and has occurred in nontraumatic cardiac arrest resuscitation^11^. The initial 160 mmHg internal pressure goal was chosen because from 16 mL/160 mmHg upward, every additional milliliter of filling volume increases the internal pressure by about 10 mmHg (an easily remembered amount), until the balloon’s maximum filling volume of 24 mL is reached, which corresponds to a target pressure of 240 mmHg. The initial 160 mmHg and corresponding 16 mL filling volume also serve as a good starting point for titration, since the balloon diameter of about 27 mm with 16 mL filling volume occludes most patients’ intrathoracic descending aortas at the level of the diaphragm. We advise against aiming directly for 240 mmHg, because the compliance curve of the balloon wedged within a noncompliant vessel is steep and titration difficult, with a considerable risk of inadvertently exposing the balloon and the vessel to excessive pressures. We do not know at exactly what internal pressure the balloon will rupture, but for safety reasons this is well below the pressure at which a rupture of vessels occurs (according to the manufacturer), and this also depends on the surrounding, external pressure counterbalancing the outward force of an overinflated balloon. For the ER-REBOA catheter, internal balloon pressures above 240–250 mmHg are never necessary in the clinical context; at full inflation, the internal pressure is below 175 mmHg without external pressure. For that reason, external and back pressure are at least 75 mmHg if internal pressure in the balloon exceeds 250 mmHg, a blood pressure level at which the risk of REBOA outweighs the potential benefits for patients.

After stabilization of the patient, deflation of the REBOA balloon should be guided by blood pressure and clinical signs of successful resuscitation. Sudden deflation of the balloon can lead to hemodynamic instability or even rearrest through the sudden fall in peripheral resistance and rapid reperfusion with washout of acidic blood products from the nonperfused lower parts of the body. As long as the pressure within the balloon remains higher than the proximal systolic pressure, no flow will occur distally. Once systolic pressure is higher, trickle flow will start, allowing for some degree of “partial REBOA” (incomplete vessel occlusion that allows a little perfusion distally). Unfortunately, with the COMPASS device displaying mean pressure in the balloon, it is difficult to predict exactly when change from full to partial occlusion is going to happen, but this could be done with a commonly used generic pressure transducer. In any case, it is prudent to release balloon pressure slowly while monitoring blood pressures proximal (tip) and distal (side port of introducer sheath with, e.g., a second COMPASS or other pressure transducer) to the occlusion, especially once the change from full to partial occlusion occurs. The recently launched P-REBOA-Pro device, a new model from the company Prytime, was developed to overcome this problem and allow for titrating flow rates for partial REBOA. Inversely, if blood pressure rises during resuscitation and the balloon pressure is not adjusted, “automatic” partial perfusion might occur, which is helpful especially in zone 1 REBOA.

### Limitations

The results of this bench study and our recommendations are restricted to the ER-REBOA catheter from Prytime Medical and the COMPASS Centurion device from Centurion Medical Products and cannot be easily transferred to other REBOA devices from Prytime or other companies. For example, the Coda Balloon Catheter (32 mm, 9Fr shaft, 100 cm; Cook, Lucerne, Switzerland) has a low-compliance balloon, which renders this method of measuring balloon pressure to guide inflation useless. Although we used the COMPASS device for practical reasons, any pressure-reading system (standard transducer/monitor) can be used for this purpose. In fact, for the bench calculations, we used the pressure readings from the transducers of the Biopac system, which were connected in-line with the COMPASS device (see “Methods” section).

The pulsatile flow was generated by the MEDOS DP3 pump, within a circuit lacking relevant Windkessel function. Pump parameters and clamps representing the peripheral resistance were set to achieve the desired pressures (diastolic pressure of 30 mmHg), which generated a total flow of 3.3 L/min, and 2 L in the simulated descending aorta. This might overestimate the flow generated by CPR and underestimate the systolic pressure, which in our circuit resembles more a parabola than a systolic “peak.” In a clinical study with 100 out-of-hospital cardiac arrest patients, diastolic blood pressure was about 30 mmHg^23^, which is in line with our experience in this patient population. With REBOA, pressures in the proximal aorta rise even before the vessel is occluded completely, and the same effect could be seen in our circuit. In addition, in tubes as in our circuit, and similarly in larges vessels as in the aorta, Ohm’s law applies and the flow is tightly related to the pressure difference. Therefore we conclude that our results and the rule-of-thumb recommendations are valid over a range of pressures and flows and independent of the cause of the low flow/low pressure condition (cardiac arrest or trauma).

For the rule-of-thumb algorithm, we assumed a 40 mmHg mean arterial pressure as the external surrounding pressure. If the external pressure is lower (e.g., during CPR), occlusion of the aorta would still be safely achieved by following the algorithm. Conversely, if the mean arterial pressure is substantially higher than the assumed 40 mmHg, the occlusion cannot be predicted exactly, and additional internal pressure (10–20 mmHg) needs to be added. In practice, the assumed 30 mmHg calculated back pressure should cover these smaller excess pressure differences, keeping in mind that patients with mean arterial pressure higher that 55 mmHg rarely need a fully occluding REBOA maneuver.

The “static” measurements of set 1 were made in a model that used two fixed tube diameters as the “aorta.” Compliance and stiffness of these silicone tubes might be different from human aortas. We cannot say for sure that the method will work in children with smaller vessel diameters or in patients with much enlarged aortic diameters.

Of note, almost all instructions for use of REBOA catheters describe the use of fluoroscopy to guide placement and filling of the balloon, except, to our knowledge, the ER-REBOA from Prytime Medical and the REBOA Balloon Kit from Vingmed AS (Norway), if no fluoroscopy is available. Using this method to fill the balloon might be a deviation from the proposed standard of care. We argue, and are supported by the literature^12,14–16,24,25^, that catheters are increasingly being placed outside hospitals, in settings lacking fluoroscopy facilities. Our method adds a safety feature that prevents overinflating the catheter’s balloon and associated vessel damage, helps monitor balloon filling to recognize balloon rupture and unintended deflation, and, after successful resuscitation, can guide the first step of safe deflation.

In conclusion, measurement of the internal balloon pressure of the ER-REBOA catheter (Prytime Medical) with a COMPASS device (Centurion Medical Products) may help identify the occlusion pressure in places where fluoroscopy is not available. We developed a simple rule of thumb to guide balloon inflation for frequent clinical circumstances. In principle, this could be expanded to every REBOA catheter with a high-compliance balloon, and every generic pressure-monitoring system. With continuous internal balloon pressure monitoring during occlusion, unintended deflation or balloon rupture can be recognized. After successful resuscitation, internal balloon pressure can guide the first step of deflation.

## Supplementary Information

Supplementary Information 1.

## Data Availability

The data sets used and/or analyzed during the current study are available from the corresponding author on reasonable request.

## Abbreviations

CFA: Common femoral artery
CPR: Cardiopulmonary resuscitation
ECC: Extracorporeal circulation
ECMO: Extracorporeal membrane oxygenation
REBOA: Resuscitative endovascular balloon occlusion of the aorta
